# Bioimpedance cardiography in pregnancy: A longitudinal cohort study on hemodynamic pattern and outcome

**DOI:** 10.1186/s12884-016-0918-8

**Published:** 2016-06-01

**Authors:** Martin Andreas, Lorenz Kuessel, Stefan P. Kastl, Stefan Wirth, Kathrin Gruber, Franziska Rhomberg, Fatemeh A. Gomari-Grisar, Maximilian Franz, Harald Zeisler, Michael Gottsauner-Wolf

**Affiliations:** Department of Surgery, Division of Cardiac Surgery, Medical University of Vienna, Vienna, Austria; Department of Obstetrics and Gynecology, Medical University of Vienna, Vienna, Austria; Department of Internal Medicine II, Division of Cardiology and Angiology all above, Medical University of Vienna, Austria, Vienna, Austria; Institute for Service Marketing and Tourism, University of Economics and Business, Vienna, Austria

**Keywords:** Eclampsia, HELLP, High-risk pregnancy, Hypertension in pregnancy, Preeclampsia

## Abstract

**Background:**

Pregnancy associated cardiovascular pathologies have a significant impact on outcome for mother and child. Bioimpedance cardiography may provide additional outcome-relevant information early in pregnancy and may also be used as a predictive instrument for pregnancy-associated diseases.

**Methods:**

We performed a prospective longitudinal cohort trial in an outpatient setting and included 242 pregnant women. Cardiac output and concomitant hemodynamic data were recorded from 11^th^–13^th^ week of gestation every 5^th^ week as well as at two occasions post partum employing bioimpedance cardiography.

**Results:**

Cardiac output increased during pregnancy and peaked early in the third trimester. A higher heart rate and a decreased systemic vascular resistance were accountable for the observed changes. Women who had a pregnancy-associated disease during a previous pregnancy or developed hypertension or preeclampsia had a significantly increased cardiac output early in pregnancy. Furthermore, an effect of cardiac output on birthweight was found in healthy pregnancies and could be confirmed with multiple linear regression analysis.

**Conclusions:**

Cardiovascular adaptation during pregnancy is characterized by distinct pattern described herein. These may be altered in women at risk for preeclampsia or reduced birthweigth. The assessment of cardiac parameters by bioimpedance cardiography could be performed at low costs without additional risks.

## Background

Hemodynamic adaptations during normal pregnancy enable the female body to comply with increased metabolic demands to fulfill fetal requirements. Blood pressure decreases during pregnancy as a consequence of vascular dilatation, which in turn leads to an increase of cardiac output due to an increased heart rate and a higher stroke volume [1, 2]. These changes are paralleled by an increase of cardiac biomarkers like NT-proBNP early during pregnancy [3].

However, this “stress test” may reveal underlying cardiovascular diseases [4]. Furthermore, distinct pathologies occur during pregnancy with potential severe sequels for mother and child [5]. Persistent hypertension occurs in 12 – 22 % of all pregnancies and may be associated with preeclampsia [6]. Eclampsia, HELLP-syndrome and peripartum cardiomyopathy occur more rarely. Increased vascular resistance, high blood pressure, pathologic endothelial function and lower plasma volume characterize preeclampsia, which has an estimated incidence up to 5 % [7]. Diagnostic criteria for preeclampsia are the onset of hypertension after the 20^th^ week of gestation, measured on two different occasions and proteinuria [8].

The assessment of cardiac output and systemic vascular resistance adds significant information to blood pressure and heart rate. As a key finding in prior trials, an increased cardiac output could be detected as early as in the 10^th^–14^th^ week of gestation in women who developed preeclampsia or gestational hypertension later in pregnancy [9]. Thus, the early and significant difference in cardiac output compared to healthy pregnancies may be used as a diagnostic tool for early identification of women at risk for preeclampsia or gestational hypertension. Furthermore, a decrease of cardiac output and an increase of blood pressure during the clinical onset of preeclampsia was reported [10]. This crossover was not detected in patients with gestational hypertension and supports the hyperdynamic disease model for preeclampsia.

The gold standard for the assessment of cardiac output is thermodilution using a cardiac catheter [11]. Although this is a suitable method for intensive care patients, it cannot be applied in an outpatient setting for repeated measurements in pregnant women. Echocardiography was previously applied to elucidate cardiovascular adaptations during pregnancy [12]. However, measurements are time consuming, technical demanding and investigator–dependent. Therefore, it gained no widespread use for screening purposes in an outpatient setting.

We decided to perform a prospective trial in order to establish the applicability of bioimpedance cardiography for the assessment of cardiovascular parameters during pregnancy [13]. Several authors reported the reliable application of this technique in healthy pregnant women and patients suffering from preeclampsia, but large prospective cohort trials are scarce [14–18].

## Methods

Between 2006 and 2009, 242 pregnant women were included in this prospective cohort study after providing written informed consent. A high number of women were included to evaluate also disease processes not primarily present and occurring throughout the pregnancy. The local ethics committee approved the study protocol (EK 619/2006). We measured cardiovascular parameters noninvasively by monitoring the tissue impedance of distinct thoracic regions [18]. An impedance cardiography monitor was used for non-invasive measurement of cardiac output, heart rate, blood pressure, systemic vascular resistance and stroke volume (ICG hemodynamic measurement, Philips Medical Systems, Andover, MA, USA; www.medical.philips.com). Two pairs of electrodes were placed on the neck and two pairs were placed on the trunk, respectively. Every measurement was performed in a quiet room after a resting period of 10 min. The automatic assessment and data printout were performed after stable readings could have been achieved. To estimate the hemodynamic influence of a growing fetus compressing the Vena cava in a supine position, every measurement was performed in supine as well as in side position. Cardiac output and not cardiac index was chosen for all analysis hence previous results indicated a low correlation of cardiac output to body surface area during pregnancy [19].

Women were asked to participate in this study at their routine visit for the first ultrasound control at the outpatient department of the obstetrics and gynecology clinic usually performed between the 11^th^–13^th^ week of gestation. Patients with pre-existing diabetes, cardiac disease (e.g. previous surgery, significant aortic stenosis and other preexisting anatomic lesions) or intravenous drug abuse were not included. Every visit included medical history assessment, a physical examination to diagnose symptoms of preeclampsia and other pregnancy related diseases, an ECG and the measurement of cardiac output, blood pressure, heart rate, systemic vascular resistance and stroke volume. Cardiac output and concomitant data were recorded at the 11^th^–13^th^, 14^th^–17^th^, 18^th^–22^nd^, 23^rd^–27^th^, 28^th^–32^nd^, 33^rd^–36^th^ and after the 37^th^ week of gestation as well as six weeks and six months post partum. Every onset of a pregnancy related disease was recorded, evaluated and treated according to current guidelines [8]. Preeclampsia was defined as the new onset of hypertension and proteinuria in human pregnancy after the 20^th^ week of gestation [8]. The gestational age was calculated from the first day of the last menstrual bleeding and was corrected according to sonographic measurements (fetal crown to rump length).

### Patient grouping

**Fig. 1:**
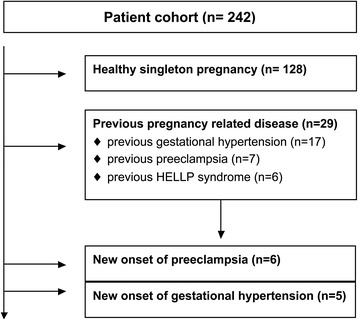
Flowchart of studied patient groups

A flow chart was added to depict final patient groups for the analysis performed in this study (Fig. 1). Pre-existing illnesses comprised hypertension (*n* = 28) and thyroid gland disease (*n* = 18; hypothyreosis 14, hyperthyreosis 4). Nine patients suffered from gestational diabetes mellitus during a previous pregnancy. Nine patients had a family history of preeclampsia. Due to a low number or heterogonous groups, patients with the aforementioned characteristics were not analyzed as specific group. However, patients with previous pregnancy related hypertension (*n* = 17), previous preeclampsia (*n* = 7) or previous HELLP syndrome (*n* = 6) were grouped (*n* = 29) and compared to the healthy control group.

During the pregnancy under investigation, patients were diagnosed with preeclampsia (*n* = 6), new onset of hypertension (*n* = 5), gestational diabetes mellitus (*n* = 12) and HELLP syndrome (*n* = 2). In addition, abnormal Doppler (*n* = 4) and preterm labor (*n* = 9) were recorded.

All women without any previous or current relevant disease described herein were classified as healthy and grouped for analysis (*n* = 128). However, six women had a twin pregnancy and were, although healthy, not included in the “healthy” group due to changed hemodynamic parameters in twin pregnancies.

### Statistical analysis

Data sets were descriptively analyzed and are presented as means ± SD. For comparison of data, sets were tested for normal distribution (Shapiro-Wilk test). To compare outcome parameters between groups of healthy and pathologic pregnancies, an analysis of variance was performed. The Pearson correlation coefficient was calculated to analyze relationships. Time points were compared pair-wise with a Sidak correction for the significance level due to the multiple comparisons applied herein. A multiple linear regression was performed to assess the effect of cardiac output on birthweight including maternal age, body mass index and smoking habit. *p*-values (two-sided) lower than 0.05 were considered statistically significant. Statistical analysis was performed with SPSS 21 (SPSS Inc., Chicago, Illinois, USA). Figures were calculated and drawn by applying the spline function. Single data points were printed for comparisons with a low number of measurements.

## Results

**Fig. 2:**
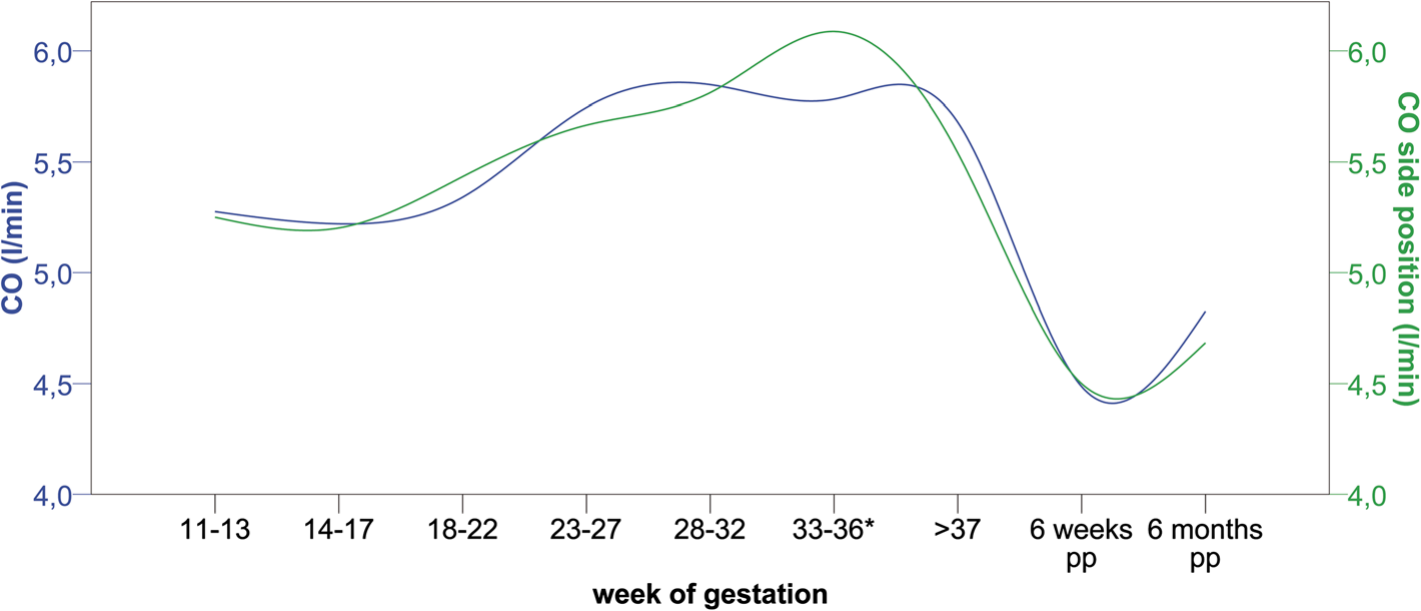
Cardiac output during healthy pregnancy (*n* = 128). Caption: CO: cardiac output; blue: supine position; green: side position; *: *p* < 0.05; pp: post-partum

Demographic characteristics of healthy and diseased pregnancies are shown in Table 1. We performed all measurements in the supine and side position to identify position dependent influences on hemodynamic parameters (Table 2, Fig. 2). A significant difference in cardiac output and stroke volume between supine and side position in healthy women (*n* = 128) was only present in the 33^rd^–36^th^ week of gestation. A significant and high correlation of cardiovascular parameters between body positions was present throughout pregnancy (Table 2). All further comparisons were performed with measurements in the side position.

**Table 1:** Demographics, pregnancy characteristics and risk factors

Group		Healthy	Prev. HTN/PRE	*p*-value	HTN/PRE	*p*-value
Demographics						
Age	*years*	32 ± 6	35 ± 5	0.010	34 ± 6	
Height	*cm*	164 ± 16	165 ± 7		166 ± 6	
Weight	*kg*	63 ± 13	78 ± 19	<0.001	80 ± 24	<0.001
BMI	*kg/m* ^*2*^	23.2 ± 4.3	29.0 ± 7.5	<0.001	28.6 ± 7.9	<0.001
Pregnancy details						
Pregnancy	*n*	2 ± 2	3 ± 2		3 ± 1	
Birthweight	*g*	3334 ± 539	2351 ± 1314	<0.001	2418 ± 1076	<0.001
Gestational week of birth	*week*	39 ± 2	35 ± 7	<0.001	36 ± 4	0.002
Previous diseases/risks						
Kidney disease	*%*	0	0		0	
Thyroid gland disease	*%*	0	3	0.035	9	0.001
Hypertension	*%*	0	38	<0.001	27	<0.001
Smoking	*%*	26	17		11	

Caption: Prev. HTN/PRE: patients with a previous pregnancy related hypertension or preeclampsia are presented in this group; HTN/PRE: Patients who developed hypertension or preeclampsia during the pregnancy under investigation are grouped herein; *p*-values are calculated against healthy group

**Table 2:** Hemodynamic parameters in healthy pregnancy (*n* = 128)

	*week*	11^th^-13^th^	14^th^-17^th^	18^th^-22^nd^	23^rd^-27^th^	28^th^-32^nd^	33^rd^-36^th^	37^th^-40^th^	6^th^ week	6^th^ month
CO	*l/min*	5.3 ± 1.1	5.2 ± 1.1	5.3 ± 1.0	5.8 ± 1.2	5.8 ± 1.1	5.8 ± 1.3	5.8 ± 1.1	4.5 ± 0.7	4.8 ± 1.1
CO-SP	*l/min*	5.3 ± 1.2	5.2 ± 1.2	5.4 ± 1.0	5.7 ± 1.2	5.8 ± 1.2	6.1 ± 1.3	5.5 ± 1.1	4.5 ± 0.8	4.7 ± 1.0
*p*-value							0.021			
Correlation	*Pearson*	0.80	0.85	0.80	0.85	0.78	0.78	0.74	0.62	0.69
HR	*beats/min*	78 ± 10	81 ± 11	83 ± 11	87 ± 16	94 ± 13	93 ± 13	90 ± 13	76 ± 11	76 ± 10
HR-SP	*beats/min*	78 ± 10	81 ± 10	82 ± 11	88 ± 11	93 ± 13	91 ± 12	89 ± 15	74 ± 10	76 ± 10
*p*-value										
Correlation	*Pearson*	0.60	0.86	0.78	0.72	0.66	0.57	0.54	0.87	0.70
BP-sys	*mmHg*	107 ± 10	102 ± 9	102 ± 9	103 ± 9	105 ± 9	105 ± 7	107 ± 10	103 ± 8	106 ± 7
BP-dia	*mmHg*	71 ± 8	69 ± 7	69 ± 7	71 ± 6	72 ± 8	74 ± 6	76 ± 8	71 ± 6	74 ± 7
SVR	*dyn · s/cm* ^*5*^	1245 ± 245	1218 ± 241	1190 ± 214	1176 ± 253	1116 ± 206	1169 ± 282	1220 ± 251	1414 ± 230	1378 ± 288
SVR-SP	*dyn · s/cm* ^*5*^	1292 ± 233	1255 ± 256	1205 ± 284	1174 ± 335	1139 ± 234	1097 ± 224	1244 ± 253	1422 ± 274	1453 ± 312
*p*-value		0.036					0.009			
Correlation	*Pearson*	0.76	0.79	0.66	0.85	0.74	0.79	0.71	0.71	0.70
SV	*l/min*	69 ± 13	66 ± 14	67 ± 13	65 ± 14	64 ± 13	63 ± 16	65 ± 14	62 ± 14	66 ± 18
SV-SP	*l/min*	67 ± 13	66 ± 15	67 ± 13	66 ± 14	64 ± 14	68 ± 14	66 ± 16	62 ± 12	62 ± 15
*p*-value							<0.001			
Correlation	*Pearson*	0.75	0.84	0.65	0.84	0.76	0.88	0.73	0.72	0.85

*CO* cardiac output, *CO-SP* cardiac output in side position, *HR* heart rate, *HR*-*SP* heart rate in side position, *BP-sys* systolic blood pressure, *BP*-*dia* diastolic blood pressure, *SVR* systemic vascular resistance, *SVR-SP* systemic vascular resistance in side position, *SV* stroke volume, *SV-SP* stroke volume in side position; only significant *p*-values are shown are shown for differences between the two positions. All correlations are significant

**Fig. 3:**
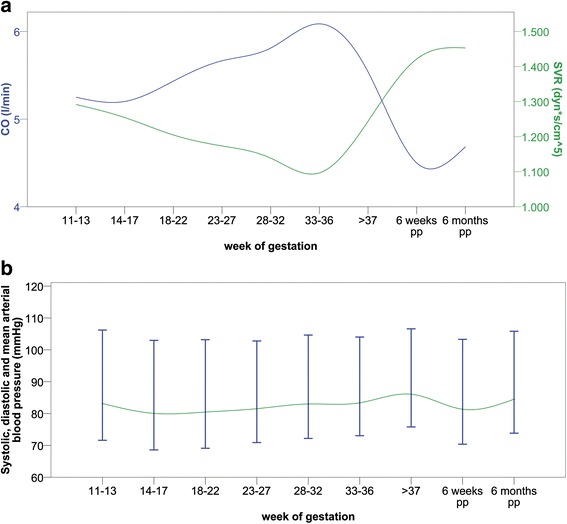
Hemodynamic parameters in healthy pregnancy (*n* = 128). Caption: **a**) CO: cardiac output; SVR: systemic vascular resistance; **b**) blood pressure; pp: post-partum

Cardiac output in the 33^rd^–36^th^ week of gestation was significantly increased compared to 11^th^–13^th^ week (*p* = 0.008) and compared to post-delivery (Fig. 3a; *p* = 0.001). Heart rate was significantly increased from the 23^rd^ week of gestation compared to 11^th^–13^th^ week (*p* < 0.001) and compared to post-delivery (Table 2; *p* = 0.002). Systolic blood pressure and stroke volume showed no significant difference throughout pregnancy and after delivery (Table 2, Fig. 3b). However, diastolic blood pressure was significantly higher after the 34^th^ week of gestation compared to 14^th^ – 22^nd^ week of gestation (Table 2, Fig. 3b; *p* < 0.02). Furthermore, systemic vascular resistance was significantly lower in the 33^rd^–36^th^ week of gestation compared to 11^th^–13^th^ week (*p* = 0.009) and compared to post-delivery (Fig. 3a; *p* = 0.006).

**Fig. 4:**
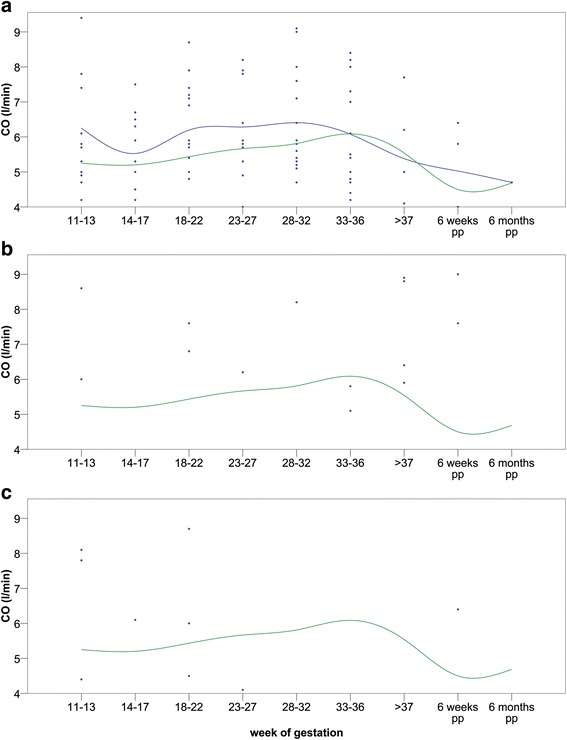
Cardiac output in healthy women and patients during pregnancy. Caption: **a**) Patients with previous pregnancy associated hypertension or preeclampsia (blue) compared to healthy women (green); **b**) patients developing hypertension during the current pregnancy (blue) compared to healthy women (green); **c**) patients developing preeclampsia (blue) compared to healthy women (green); CO: cardiac output; pp: post-partum

Women with preeclampsia, HELLP syndrome or hypertension in a previous pregnancy were grouped and analyzed (Table 3, Fig. 4a; *n* = 29). This group had significantly increased cardiac output and heart rate in the first months and a significantly increased blood pressure throughout pregnancy. Eleven patients had a previously diagnosed hypertension, two patients developed preeclampsia, two patients developed HELLP syndrome, two patients had an abnormal Doppler and two patients suffered from preterm delivery.

**Table 3:** Previous pregnancy related disease (*n* = 29) and healthy controls (*n* = 128)

week		11^th^-13^th^	14^th^-17^th^	18^th^-22^nd^	23^rd^-27^th^	28^th^-32^nd^	33^rd^-36^th^	37^th^-40^th^	6^th^ week	6^th^ month
CO	prev. dis.	6.3 ± 2.2	5.5 ± 1.1	6.2 ± 1.5	6.3 ± 1.4	6.4 ± 1.7	6.1 ± 1.5	5.4 ± 1.6	5.0 ± 1.3	4.7 ± 0
*l/min*	healthy	5.3 ± 1.2	5.2 ± 1.2	5.4 ± 1.0	5.7 ± 1.2	5.8 ± 1.2	6.1 ± 1.3	5.5 ± 1.1	4.5 ± 0.8	4.7 ± 1.0
p-value		0.017		0.012						
HR	prev. dis.	85 ± 9	85 ± 14	90 ± 8	91 ± 14	92 ± 13	91 ± 10	100 ± 10	72 ± 11	82 ± 0
*beats/min*	healthy	79 ± 10	81 ± 11	83 ± 11	88 ± 12	94 ± 14	91 ± 14	89 ± 15	74 ± 10	76 ± 10
p-value		0.021		0.003						
BP-sys	prev. dis.	124 ± 15	117 ± 16	119 ± 13	115 ± 11	112 ± 11	115 ± 18	112 ± 14	113 ± 5	96 ± 0
*mmHg*	healthy	107 ± 11	103 ± 7	104 ± 8	102 ± 10	104 ± 10	104 ± 9	107 ± 10	103 ± 7	106 ± 7
p-value		<0.001	<0.001	<0.001	<0.001	0.009	0.003		0.028	
BP-dia	prev. dis.	85 ± 21	80 ± 11	79 ± 10	79 ± 9	76 ± 8	79 ± 11	81 ± 9	76 ± 6	67 ± 0
*mmHg*	healthy	73 ± 8	69 ± 7	70 ± 7	71 ± 7	72 ± 9	73 ± 8	76 ± 8	70 ± 7	74 ± 7
p-value		<0.001	<0.001	<0.001	0.001	0.064	0.035			
SVR	prev. dis.	1444 ± 698	1389 ± 356	1257 ± 469	1170 ± 292	1093 ± 248	1222 ± 360	1422 ± 447	1400 ± 271	1260 ± 0
*dyn · sec/cm* ^*5*^	healthy	1292 ± 233	1255 ± 256	1205 ± 284	1174 ± 335	1139 ± 234	1097 ± 224	1244 ± 253	1422 ± 274	1453 ± 312
*p*-value										
SV	prev. dis.	74 ± 22	66 ± 16	70 ± 16	71 ± 16	72 ± 14	69 ± 14	58 ± 16	70 ± 15	63 ± 0
*l/min*	healthy	67 ± 13	66 ± 15	67 ± 13	66 ± 14	64 ± 14	68 ± 14	66 ± 16	62 ± 12	62 ± 15
*p*-value						0.063				

*CO* cardiac output, *HR* heart rate, *BP-sys*: systolic blood pressure, *BP-dia*: diastolic blood pressure, *SVR* systemic vascular resistance, *SV* stroke volume; all measurements are performed in side position, only significant and borderline significant *p*-values are shown

Patients with a new onset of pregnancy related hypertension during this pregnancy had a significantly increased cardiac output due to an increased stroke volume compared to the healthy group (Fig. 4b). Patients who developed preeclampsia had a significantly elevated blood pressure throughout pregnancy (*p* < 0.01). In addition, cardiac output was significantly increased from 5.2 to 6.8 l/min at 11^th^–13^th^ week of gestation compared to the healthy group due to an increased stroke volume (Fig. 4c; *p* = 0.04). Cardiac output was no longer increased from the 14^th^ week of gestation, but systemic vascular resistance was significantly increased (1650 ± 665 dyn · s/cm^5^ compared to 1255 ± 256 dyn · s/cm^5^; *p* < 0.05).

### Fetal outcome in healthy pregnant women

A correlation analysis of cardiac output and systemic vascular resistance with birthweight in healthy women revealed a positive correlation for cardiac output and a negative correlation for systemic vascular resistance. The Pearson correlation coefficient for cardiac output was 0.32 (*p* = 0.014), 0.42 (*p* = 0.006) and 0.32 (*p* = 0.058) for the 23^rd^–27^th^, 28^th^–32^nd^ and 33^rd^–37^th^ week of gestation, respectively. Concordantly, the Pearson correlation coefficient for systemic vascular resistance was −0.41 (*p* = 0.004), −0.33 (*p* = 0.033) and −0.31 (*p* = 0.061) for the 23^rd^–27^th^, 28^th^–32^nd^ and 33^rd^–37^th^ week of gestation, respectively. A multiple linear regression analysis including cardiac output, maternal age, body mass index and smoking habit revealed cardiac output as the only independent predictor for birthweight (*p* = 0.032 and *p* = 0.028 for the 23^rd^–27^th^ and the 28^th^–32^nd^ week of gestation, respectively). Furthermore, birthweight and gestational age were significantly reduced in hypertensive pregnancies to 2548 ± 903 g at 37 ± 5 weeks compared to 3308 ± 554 g at 39 ± 2 weeks in healthy pregnancies (*p* < 0.001 for birthweight and *p* = 0.03 for gestational age).

## Discussion

The assessment of cardiovascular parameters utilizing bioimpedance cardiography during pregnancy was performed in a number of previous trials and showed reliable results [15–18, 20, 21]. We present a large cohort study with longitudinal measurements in different patient positions compared to previous trials [22]. The influence of body position on maternal hemodynamic parameters in healthy pregnancies was assessed and a significant reduction of cardiac output due to a reduced stroke volume was observed at the 33^rd^–36^th^ week of gestation in supine position [14]. The significant influence of body position on cardiac output in the third trimester of pregnancy is in accordance with previous findings [23]. However, our data prove that body position is only relevant to cardiac parameters towards the end of pregnancy. However, we decided to use hemodynamic data acquired in the side position for all further comparisons described herein.

Cardiac output increased constantly towards the 35^th^ week of gestation in healthy pregnancies, which is concordant with previous results [1, 2]. However, stroke volume did not change significantly during healthy pregnancy. We conclude therefore, that stroke volume was not responsible for the increased cardiac output. Our data suggest that systemic vascular resistance, which was already decreased at inclusion and further decreased until the 24^th^ week of gestation, drives cardiac output by reduced afterload and a higher heart rate. Compared to the results of San-Frutos et al., systemic vascular resistance was relatively stable thereafter until close to delivery and showed no increase after the 24^th^ week of gestation [17]. This difference may be due to our higher number of observations and different measurement equipment. Our data are further supported by Bosio et. al., who measured hemodynamic parameters by echocardiography and showed a similar time course of systemic vascular resistance [9]. Heart rate also increased continuously from the 24^th^ week on and the diastolic blood pressure increased close to delivery. Both findings are in good agreement with the data presented by Volman et al., but the distinct pattern of cardiac output during pregnancy is still under debate [1]. A recent meta-analysis by Meah et al., concluding previous trials, highlighted the ongoing discussion regarding cardiac output especially in the third trimester [22]. Our results add to this discussion and support the results of the meta-analysis regarding a drop of cardiac output close to term. However, we did not exhibit an earlier drop of cardiac output at the end of the second trimester, which had been suggested based on the meta-analysis, but was not confirmed or rejected previously with data from a large cohort trial [22]. We therefore believe that our results are a relevant addition to the current data regarding cardiac output in healthy pregnancies.

In addition to the healthy population, special groups of patients presenting a cardiovascular or pregnancy associated pathology or are at risk to develop one of these disorders were included. Women with previous pregnancy associated hypertension or preeclampsia had a different hemodynamic profile throughout pregnancy compared to healthy pregnancies without previous pathologies. A long-lasting effect on cardiovascular compliance after pregnancy was previously described in healthy pregnancies [24]. This may also be true after pathologic pregnancies. Furthermore, underlying risk factors inducing the hemodynamic alterations may still be present in these women. Significant adverse outcomes are reported in this group [25]. We suggest that a previous pregnancy associated disease may be a risk factor for adverse hemodynamic behavior in a consecutive pregnancy. The screening for disturbed cardiovascular adaptation during pregnancy may therefore be beneficial for women with a history of pregnancy-associated disease.

In addition, we were able to measure cardiac parameters prior to disease onset and could provide insights in early adverse alterations of hemodynamic function. An increased cardiac output during early pathologic pregnancy was previously described and supports the hypothesis of a hyperdynamic hemodynamic state inducing preeclampsia [9, 26]. The increase of systemic vascular resistance later in pregnancy leads to hypertension and a reduction of cardiac output [10]. Bioimpedance cardiography may provide additional information for patients at risk for preeclampsia. However, distinct hemodynamic characteristics of these patient groups should further be specified in a multicenter trial.

Interestingly, cardiac output showed an impact on birthweight in healthy pregnancies. Altered cardiovascular conditions in pregnancies with fetal growth restrictions were previously reported and are in good accordance with our data [2, 21, 27, 28]. In addition to previous trials, our results indicate that these changes may also appear early in otherwise healthy pregnancies and can be measured by bioimpedance cardiography [20]. These findings should also be reevaluated in larger clinical trials. Patients identified to be “at risk” by bioimpedance cardiography may undergo further cardiovascular workup and increased clinical controls until birth.

Gestational age and birthweight were also significantly decreased in hypertensive women. No significant difference between treated and untreated women could be detected. Previous trials reported conflicting results whether chronic hypertension impacts fetal outcome [29, 30]. However, the difference in birthweight seems much more pronounced than in gestational age, which may indicate a growth restriction in these pathologies independent of gestational age.

### Limitations

Although this prospective cohort trial included a high number of pregnant women compared to other trials, the number of patients diagnosed with preeclampsia and new onset of hypertension is limited. Therefore, the findings in these subgroups have to be interpreted with caution. Further, not all patients participated in all measurement time points. Some groups were not analysed due to the limited number of patients (thyroid disease, gestational diabetes and twin pregnancies). Measurements in pathologic groups were not analyzed in relation to the body mass index due to the limited number of observations.

## Conclusion

In conclusion, cardiovascular adaptation during pregnancy is altered in women at risk for preeclampsia or reduced birthweigth. Distinct hemodynamic pattern were present early in pregnancy and could be assessed by bioimpedance cardiography at low costs without additional risk.

## Abbreviations

ECG, electrocardiogram; HELLP, Hemolysis, Elevated Liver enzymes, Low Platelet count; ICG, impedance cardiography; SD, standard deviation; SPSS, Statistical Package for the Social Sciences
